# Cognitive‐behavioral therapy in the time of coronavirus: Clinician tips for working with eating disorders via telehealth when face‐to‐face meetings are not possible

**DOI:** 10.1002/eat.23289

**Published:** 2020-05-08

**Authors:** Glenn Waller, Matthew Pugh, Sandra Mulkens, Elana Moore, Victoria A. Mountford, Jacqueline Carter, Amy Wicksteed, Aryel Maharaj, Tracey D. Wade, Lucene Wisniewski, Nicholas R. Farrell, Bronwyn Raykos, Susanne Jorgensen, Jane Evans, Jennifer J. Thomas, Ivana Osenk, Carolyn Paddock, Brittany Bohrer, Kristen Anderson, Hannah Turner, Tom Hildebrandt, Nikos Xanidis, Vera Smit

**Affiliations:** ^1^ Department of Psychology University of Sheffield Sheffield UK; ^2^ Central and North West London NHS Foundation Trust London UK; ^3^ University of Maastricht Maastricht The Netherlands; ^4^ South Yorkshire Eating Disorders Association Sheffield UK; ^5^ Maudsley Health, Abu Dhabi, and South London and Maudsley NHS Trust London UK; ^6^ Memorial University of Newfoundland St. Johns Newfoundland Canada; ^7^ NHS Specialist Eating Disorders Service Sheffield UK; ^8^ University of Toronto Toronto Ontario Canada; ^9^ Blackbird Initiative, Órama Institute, Flinders University Adelaide South Australia Australia; ^10^ Center for Evidence Based Treatment Shaker Heights Ohio USA; ^11^ Rogers Behavioral Health Oconomowoc Wisconsin USA; ^12^ Centre for Clinical Interventions Perth Western Australia Australia; ^13^ Dorset All Age Eating Disorders Service Bournemouth UK; ^14^ Eating Disorder Service, Greater Manchester Mental Health NHS Trust Manchester UK; ^15^ Department of Psychiatry Harvard Medical School Boston Massachusetts USA; ^16^ Bedfordshire and Luton Community Eating Disorders Service Luton UK; ^17^ UCSD Eating Disorders Center for Treatment and Research San Diego California USA; ^18^ Chicago Center for Evidence‐Based Treatment Chicago Illinois USA; ^19^ Southern NHS Foundation Trust Southampton UK; ^20^ Mount Sinai School of Medicine New York New York USA; ^21^ Lanarkshire NHS CAMHS Eating Disorders Service Lanarkshire UK; ^22^ Department of Eating Disorders GZ Centraal Hilversum The Netherlands

**Keywords:** cognitive‐behavioral therapy, coronavirus, COVID‐19, eating disorders, psychotherapy, telehealth

## Abstract

**Objective:**

The coronavirus pandemic has led to a dramatically different way of working for many therapists working with eating disorders, where telehealth has suddenly become the norm. However, many clinicians feel ill equipped to deliver therapy via telehealth, while adhering to evidence‐based interventions. This article draws together clinician experiences of the issues that should be attended to, and how to address them within a telehealth framework.

**Method:**

Seventy clinical colleagues of the authors were emailed and invited to share their concerns online about how to deliver cognitive‐behavioral therapy for eating disorders (CBT‐ED) via telehealth, and how to adapt clinical practice to deal with the problems that they and others had encountered. After 96 hr, all the suggestions that had been shared by 22 clinicians were collated to provide timely advice for other clinicians.

**Results:**

A range of themes emerged from the online discussion. A large proportion were general clinical and practical domains (patient and therapist concerns about telehealth; technical issues in implementing telehealth; changes in the environment), but there were also specific considerations and clinical recommendations about the delivery of CBT‐ED methods.

**Discussion:**

Through interaction and sharing of ideas, clinicians across the world produced a substantial number of recommendations about how to use telehealth to work with people with eating disorders while remaining on track with evidence‐based practice. These are shared to assist clinicians over the period of changed practice.

## 1 INTRODUCTION

On March 11, 2020, a coronavirus disease pandemic was declared by the World Health Organisation (WHO). While this new virus (hereafter referred to as COVID‐19) had first been identified several months earlier, this declaration marked recognition that it was showing rapid growth across many countries. Many governments focused their efforts on reducing the risk of cross‐infection, recommending and enforcing social distancing (physical distance between individuals, banning of meetings, cancellation of sporting fixtures, and closing of schools, universities, shops, bars, restaurants, and workplaces).

This level of social isolation has had many social, economic and health impacts. Among those impacts, many clinicians working with outpatients with eating disorders have had to transfer from a norm of face‐to‐face practice to delivering real‐time treatment via videoconferencing programs (ideally) or telephones, known as telehealth. For many interventions, and particularly for psychological therapies, this change to telehealth and the wider public concerns about the impact of COVID‐19 have required us to develop new ways of working at very short notice. This change in practice means that there is a need for substantial adaptability on the part of therapists, patients and carers alike. At this stage in the spread of COVID‐19 and our efforts to contain and reduce it, the primary question that we are facing is: what lessons do outpatient therapists need to learn about how to adapt to telehealth methods?

Telehealth has been developed and shown to be effective broadly (Backhaus et al., 2012). It has also been demonstrated that its effects can be equivalent to those of face‐to‐face therapy in specific areas of mental health, such as post‐traumatic stress disorder (Acierno et al., 2016, 2017; Morland et al., 2014, 2015; Yuen et al., 2015). However, while telehealth is not new in the field of eating disorders, its evidence base is less well developed. In part, this is because evidence‐based treatments have been predicated largely on face‐to‐face contact (e.g., monitoring risk; the weighing of patients—Waller & Mountford, 2015). There are some preliminary studies showing that telehealth can be beneficial in treating eating disorders and obesity (e.g., Abrahamsson, Ahlund, Ahrin, & Alfonsson, 2018; Anderson, Byrne, Crosby, & Le Grange, 2017; Cassin et al., 2016; Giel et al., 2015; Hamatani et al., 2019; Sockalingam et al., 2017), but fewer substantial studies that support this approach (e.g., Ertelt et al., 2011; Mitchell et al., 2008). Kazdin, Fitzsimmons‐Craft, and Wilfley (2017) have identified telehealth as requiring further study and development, though the limited evidence to date suggests we can deliver effective therapies for eating disorders at a distance with thoughtful planning and careful delivery (Sproch & Anderson, 2019).

In the case of cognitive‐behavioral therapy for eating disorders (CBT‐ED), existing evidence‐based protocols and practice (e.g., Fairburn, 2008; Thomas & Eddy, 2018; Waller, Turner, Tatham, Mountford, & Wade, 2019) mean that clinicians are used to working face‐to‐face with their clients and carers. The need to transfer to a telehealth approach with practically no notice meant that many CBT‐ED therapists needed to work out how to arrange a new way of working with their patients in just a few days. This need led to clinicians beginning to share ideas and experiences that would be of use in CBT‐ED, but which also had the potential to support other therapies in making this transition. The aim of this article is to share the ideas that were generated by this process, to make available the clinical techniques and process considerations of a number of experienced clinicians.

## 2 METHOD

This report is based on the ideas shared on an online form, where clinicians could add their experiences and ideas about how to work with eating disorders using telehealth methods. The work was not a research study, so it was not appropriate to seek ethical approval. The form was launched on March 24, 2020—13 days after the WHO had declared a coronavirus disease pandemic, and at a stage where levels of response to COVID‐19 were substantially different across countries (e.g., China was reporting a leveling off of new cases; Italy, Spain and other European countries were enforcing curfews; Australia, the United Kingdom and parts of the USA were moving in the same direction; some African countries were reporting their first cases). Thus, it was launched as a means of allowing clinicians to share strategies when the situation was moving toward telehealth.

The initial online form (a Google document, see Appendix A) was distributed to approximately 70 clinical colleagues internationally, who were known to be practicing CBT with eating disorder clients, with the request that it should be passed on to allow others to contribute. It was in English, so its uptake might have been limited among clinicians from non‐English‐speaking countries. Participants were specifically asked: “In these times of social isolation, most of us are moving to online contact/telehealth working with our eating‐disordered patients. There have been a lot of enquiries in the last few days about how to deliver CBT‐ED online, and we thought that it would be far more useful to make this a shared venture. In the table below, please provide useful suggestions and your experience of them.” Participants were asked to focus on how to develop CBT by telehealth; maintain confidentiality; and avoid commercial promotions.

After 96 hr, all the suggestions that had been shared by 22 clinicians (the authors of this article) were collated for this article. It is recognized that this is a relatively short timeframe, and that other suggestions might be forthcoming (these remain available online). However, our priority was to make these clinical ideas available to the wider clinical community while they were most potentially useful. The suggestions here came from clinicians in the UK, Canada, Australia, the USA, Abu Dhabi and the Netherlands.

## 3 RESULTS

### 3.1 Collaborative clinician guidance for working via telehealth

The following are the domains that were identified as needing consideration (headers) and the suggestions (bullet points) that clinicians made based on their recent experience and flexible application of protocols. Several existing guidelines for delivering therapy by telehealth routes were raised (see Appendix A) and seemed useful in general. However, they did not address the transitions that clinicians were going through in the current circumstances. Therefore, clinician experience was used to develop the following domains: patient and therapist concerns about telehealth; technical issues in implementing telehealth; changes in the environment; and implementing specific CBT‐ED methods remotely.

Local employment, supervisory, reimbursement and regulatory frameworks were also raised as meriting attention. Obviously, clinicians should alert supervisors and employers to any such change in therapy delivery method. Local or wider clinician peer groups to share ideas and methods are also likely to be useful. Where healthcare is covered by insurance, the eligibility of telehealth sessions for reimbursement should be checked, to ensure that the patients in not presented with an unexpected charge for their psychological therapy. State or national guidelines should be considered, appropriate to where the clinician is based. For example, the American Telemedicine Association Guidelines (Turvey et al., 2013) should be considered when working in the USA. Issues of working across geographical boundaries might also need to be reviewed (e.g., a clinician might work appropriately in their own state or country, but their license might not automatically extend to undertaking the same work when the patient is in a different regulatory area). Similarly, the web platform to be used needs to be compliant with both employer and licensure regulations.

We should remember that the patients who we are seeing are likely to be having the same experience of the changed circumstances as ourselves and most other people, though their experience is likely to be made more complex by the interaction with their existing levels of anxiety, coping mechanisms and control issues, Therefore we should monitor the patient's experience routinely, to ensure that we can focus on both risk and the delivery of CBT‐ED. Part of our role is likely to be helping the patient to manage their anxiety (e.g., normalizing it in the context of externally driven uncertainty and loss of control) or to address it directly (e.g., ensuring that patients understand that denying the danger of going to the gym actually adds to their risk). In cases where emergency interventions might be needed (e.g., suicidality expressed while in an online session), they should be responded to appropriately (e.g., contacting emergency services while the patient is on the line). However, as always, we should consider whether any such threat is a means of communicating distress to the therapist and respond accordingly if that appears to be the case.

It is noteworthy that the clinical experiences and suggestions that emerged were partly specific to CBT‐ED (the starting point for this collaboration), but that the majority were applicable to a very wide range of therapies. Therefore, to ensure that this generalizability is emphasized, the therapy‐specific suggestions are placed after the more generic ones. Furthermore, it was evident that clinicians were sometimes both delivering psychological treatments via telehealth and managing medication, and this dual role should be considered where appropriate. Finally, it is important to remember that the issues and suggestions raised here are impressionistic, based on therapists' own experiences and their reports of patient experiences. Therefore, these should be used as possible avenues for clinicians to explore with their patients, rather than being viewed as being based on more robust evidence.

### 3.2 Patient and therapist concerns about telehealth approaches

It is clearly critical to engage the patient in undertaking therapy online, particularly if they thought that they were going to receive face‐to‐face treatment. However, we have found that addressing the points below assist in that. Appendix B provides an outline letter regarding online delivery of CBT‐ED, to help the patient to understand and engage in the process. The letter can easily be adapted to other psychological therapies, of course.

*The patient might see the telehealth approach as “second‐best”*. The following concerns are largely ones that therapists raised as potentially being issues for the patient. Thus, they are hypothetical, but ones where the clinician needs to be prepared as the current Coronavirus situation continues and develops, in case they are raised by patients.

- The therapist should stress that it is “business as usual” in therapy terms:
  - use the protocol explicitly; continue to maintain key elements (e.g., agenda‐setting, monitoring progress, behavioral change, maintaining boundaries),
  - remain professional in dress, timekeeping,
  - stay on track (e.g., no distraction from the telephone, checking email).
- If the patient has already started face‐to‐face therapy before moving to telehealth, this can be framed as a positive shift of responsibility to the patient.
- If the patient asks to suspend therapy when they hear that the therapy will be online, the first step should be to explore their concerns and predictions and whether they can be addressed within therapy. It can be useful to ask the patient to experiment with online now and if that does not work then they can try face‐to‐face later, so they lose nothing but might get better earlier. This can become a behavioral experiment where predictions are tested.
- Ensure that the therapy does not shift into a pattern of supporting the patient in remaining unchanged. This active attitude by the therapist reduces the risk of socializing the patient into becoming passive because of the therapist's lack of belief in the possibility of change.
*Patient or therapist concerns that therapy cannot work in this way*.
- Lots of positive reinforcement for changes that the patient does make, to stress how well they are learning even in this context.
- Stress that they normally would be doing therapy 167 hr a week without the therapist being there, so the amount of time that they would be working without the therapist in the room is far more useful and important than the time with the therapist present.
- Review the experience at the end of each session (use of the session rating scale may be helpful) and reinforce the patient by pointing out that you are covering the necessary material with them.
- Consider the personal experience of therapists who are used to working via telehealth. Several therapists shared that they had already been working remotely in this way for many years and reported that they find it works well. Such therapists might be asked to coach teams and supervise clinicians who are new to delivering treatment via telehealth.

### 3.3 Technical issues

A large number of points raised related to practical issues, such as what software platform is most useful for this work, and how to ensure that work that is normally done using paper and pen can be maintained under this new way of working. A wide range of experiences and suggestions were shared. It is important for therapists to remember that the options might seem daunting if they have not been used before, but they are all relatively straightforward methods, which can be learnt quickly by clinicians who are naïve about them (as was the case for many contributing clinicians). Remember that the key is to ensure that the patient and the therapist communicate—the therapist is the key to delivery of the therapy, and the technology is just a tool to making that possible.

### 3.4 Technology experiences

- Software choices were generally positive about the use of Zoom, Facetalk, Google Meets, Vsee, and Microsoft Teams, due to experiences of reliability and quality of images.
  - Others were seen as less reliable, and insufficiently secure for this purpose (particularly Skype and Facetime),
  - Reduce public accessibility on some platforms (e.g., do not share links on social media), or there is a risk of inappropriate material being sent to you, including during sessions (e.g., “Zoom bombing”),
  - Local recommendations should be followed regarding appropriate technology to use and gaining patient consent to use those technologies,
  - Make sure that you are working within your organization's guidance regarding software use.
- Platforms that let more than two people take part (e.g., Vsee, Zoom; Google Meet) can help with family involvement, but ask people to turn off their microphones when they are not talking, to help with audibility.
- Some platforms allow you to use “talk to text” (e.g., turn on “captions” in Google Meet'), to help those who have trouble hearing. The transcription (in English, at least) is pretty good in Google Meet, but you cannot save it.
- Privacy needs to be ensured as far as possible, which might mean the patient and therapist using headphones if total privacy cannot be guaranteed (e.g., the therapist or the patient having children nearby).
- Remember to turn off alerts on your computer or phone, as they will interrupt the session, and ask the patient to do likewise.
- Turn off “assistant” devices (e.g., Alexa, Siri, Google home), as they could be recording and disseminating confidential information.
- The telephone alone can be used effectively if that is all that is available but was generally felt to be less useful than video communications, and there were concerns about patients having access to therapists' personal numbers.
  - Maybe start with audio and swiftly work up to video if the patient has concerns about seeing their own image. Alternatively, ask the patient to block looking at distressing parts of their own image at first if they cannot tolerate it, but only for a short time.
  - Discuss preferences with the patient, and experiment with what actually works better for them.
  - Turn off your number when calling, to block the patient learning your number, especially of the phone is your own. This can be done in different ways (e.g., dialing 141 before the number in the UK).
- Headsets can be useful to enhance audibility and ensure confidentiality, but they also look unnatural (the “call‐center” look), so use only if necessary.
- Ensure that you and the patient have the necessary internet/phone connections, and that costs are considered (e.g., if the patient has a very limited internet connection or phone plan).
- Where using video links, camera placement at both ends should be attended to, to ensure that both the therapist and patient can see each other as well as possible (e.g., allowing both to read non‐verbal cues).

*Communicating written material*. Overall, the importance of continuing to get the patient to self‐monitor and to report food intake and psychological status was stressed, both on a session‐by‐session basis and at the end of treatment. Similarly, it was widely suggested that psychoeducation materials should be sent to the patients. A number of recommendations were made for making this possible:

- Diaries and questionnaires can be completed as normal and scanned/sent by email, completed electronically and emailed, or completed on the patient's phone and sent in (all to arrive before the treatment session, including ahead of the first session). Platforms such as Zoom allow for sharing of documents in session.
- Resources were identified as being available for clinicians to access freely, including diaries and psychoeducation materials (see Appendix A).
- The TinyScanner app was recommended as allowing you to scan from your phone to a pdf document for emailing. Patients are reported to be very positive about this.
- Online diaries were recommended (including “Rise Up and Recover” and “Recovery Record”).
- Patient consent and secure communication methods are also important to ensure. It was agreed that a handout for patients on how to prepare for CBT‐ED via telehealth would be valuable, and this is provided in Appendix B for clinicians to use and adapt as appropriate.

### 3.5 Impact of changes in the environment

While these impacts vary with the degree of enforced or voluntary social isolation that countries implement at different stages in the Coronavirus pandemic, the following suggestions were made:

- Where the patient experiences a reduction in opportunities to exercise (e.g., closure of gyms; reduced opportunity to exercise or spend time outdoors), that can lead to concerns about potential impact on weight and fitness, as well as the loss of an anxiety management technique. In such cases, acknowledging that these outcomes are possible but are context‐dependent, and that any changes are reversible following the period of reduced activity. The attitude of “will there really be a better time to address your eating disorder than now?” can be a helpful one to communicate to the patient.
- Stress the potential positives of some of these environmental changes (e.g., the closure of gyms and the lack of access to “binge foods”), as they give the opportunity to learn that these behaviors are not essential.
  - However, where patients say that they believe that they are only engaging in fewer behaviors because those behaviors are no longer available, it is important to reframe the situation, helping them to attribute their progress to the cognitive and emotional changes that they have been working hard to bring about (e.g., “If you had really wanted to exercise, you could have done it, but instead you chose to do the exposure work that helped you face and reduce your anxiety, so well done.”).
- Supply chain problems, panic buying and limited opportunities to shop can mean that there is limited access to some foods or brands, and reduced opportunities to expose and experiment with foods. In such cases, reviewing the pattern of healthy eating and how it can be achieved flexibly is important, so that the patient is aware that nutritional needs can still be met, even if anxiety is raised to do so (e.g., trying a novel food or brand).
- A small number of patients express a fear of exposure to the COVID‐19, and its consequences. While it is important not to downplay that risk, the following should be raised in order to ensure that the patient stays on track:
  - Using online resources to explain how to eat a healthy, balanced diet (e.g., British Dietetic Association; American Academy of Nutrition and Dietetics, The Real Food Guide), which will support general health (including maintaining the immune system) to maximize ability to cope with any infection (see Appendix A),
  - Following Governmental and WHO advice regarding reducing the risk of COVID‐19 infection.
- Where the patient wants to talk about their anxiety about COVID‐19 to the exclusion of the CBT‐ED, address:
  - The importance of eating to ensure health (see above),
  - Controlling what one can, so focusing on recovery from the eating disorder,
  - Using the patient's experience of tolerating anxiety in their eating disorder treatment to manage their anxiety regarding COVID‐19,
  - The validity of their concerns about COVID‐19 can be addressed and used to support the importance of taking care of their physical health (including addressing the eating disorder).

### 3.6 CBT‐ED related techniques, and how to apply them in the telehealth context

The following are adaptations of existing CBT‐ED techniques, as described in evidence‐based approaches (e.g., Becker, Farrell, & Waller, 2019; Fairburn, 2008; Waller et al., 2007, 2019), as suggested by clinicians here.

*Eating adequately*. Obviously, changes in eating behaviors are central to the nutritional, cognitive and emotional needs of all patients with eating disorders. Patients can be concerned that particular foods and brands will not be available, and that this will mean that they cannot eat as planned. Suggestions around this area included:

- Maintain a stance of “no excuses—you can do this and can rise to the challenge”
- Enhancing the psychoeducation that we would normally deliver, stressing the importance of eating the wide range of nutrients that are needed, and that those are available in a wide range of foods:
  - Include information that is COVID‐19 specific (see Appendix A).
- Encouraging exposure to new foods and brands, to overcome specific supply issues.
- Changing food shopping patterns (e.g., different shops; using online shopping for food)
- Use existing quarantine food plans (food with an appropriate shelf life and nutritional balance—see Appendix A).

*Exposure therapy*. Exposure therapy is much easier to deliver when the individual has wider opportunities to experience unpredictable situations and to take risks that enhance their experience of anxiety and their consequent learning. Levels of isolation and inactivity clearly limit such opportunities. Apart from how to conduct mirror exposure for body image (see below), the following clinical experiences and suggestions were shared:

- Using imaginal exposure where in vivo is not possible, including getting patients to prepare plans for exposure post‐lockdown (as this will act as exposure in itself).
- Use virtual social eating opportunities (e.g., booking dates with friends to eat on webcam, or just catching up over coffee and a snack). This can also provide an opportunity to wear avoided/less concealing clothes in virtual company, if body concealment is a safety behavior.
- Consider using the therapy session as an opportunity to conduct food and/or body‐related exposure activities.
- Use more take‐out and delivery food options, where the contents and calorie contents are not known, to enhance anxiety.
- Given the tendency for binge‐eating episodes to occur in social isolation, stress to the patient that the current social climate is an opportune time to utilize cue exposure to break the association between social isolation and binge eating.

*Weighing and linking it to eating*. Open weighing is a core element of CBT‐ED (Waller & Mountford, 2015). However, we also want to ensure that weighing does not turn into checking, which might mean that we have previously advised the patient to get rid of their own scales. Therefore, we need to adapt the usual protocols to telehealth approaches. Suggestions were:

- Ask the patient to get out the weighing scales that we asked them to put away, or order a new set online, for use only in this therapeutic context. Until scales are available, then self‐measuring using specific items of clothing can be used as a substitute.
- Complete weight charts electronically based on the readings, so that you can send the patient a copy by email (an Excel version was offered by one colleague).
- Explain to the patient that scales differ and that their initial weight might differ from their last reading on the therapist's scales (and that their own scales might have greater variability).
- Get the patient weighed by other professionals if they have medical appointments.
- Ask family and carers to assist with this process if appropriate (but there needs to be a positive justification for doing so, as this could cause further difficulties).
- Ensure that the patient self‐weighs during the session, so that you can implement the process of enhancing “hot” cognitions by discussing food intake just before weighing, to enhance excessive weight predictions and consequent learning about true weight outcomes (Waller & Mountford, 2015).

There were a number of concerns about how to ensure reliable and valid weight measurements when the therapist was not present to check on the process. While there was mention of very high‐tech scales that would send in weight readings electronically, these were not expected to be available in the great majority of cases. Lower‐tech suggestions included:

- Ask the patient to video or photograph the scales to send in the reading to validate their stated weight,
- Ask family to monitor the readings to help the patient to be open about their weight, if necessary (but not automatically, and considering the potential drawbacks).

*Drawing diagrams*. As above, it is possible to share weight charts by email. However, CBT‐ED uses a number of other diagrammatic tools (energy graphs; cognitive records; pie charts; formulations). With screen sharing, these can be discussed in real time with the patients and can even be typed up or drawn in real time with some practice. Suggestions included:

- If you are using a good enough resolution video platform (see above for recommendations) that allow sharing, then you can draw the diagram and show it to the patient as you proceed. If you are doing this, remember to:
  - check that the patient can see it,
  - suggest that they copy it as you go (or scan and send it later—see above for advice on how to do this with your phone),
  - use a thick pen to draw diagrams (overcomes the problem of low‐resolution cameras).

*Body image work*. Some elements of body image work are relatively easy to set for the patient to undertake outside of the therapy session, so can be conducted as usual (e.g., psychoeducation; body checking experiments, especially if the patient has now been asked to buy scales—see above). However, others require greater adaptation to be effective via telehealth. These include the following, which clinicians suggested based on their experiences:

- Use of surveys was reported to be relatively easy to maintain, using video methods to screen‐share the outcomes (whether collated and presented by the patient or by the therapist). Whoever is distributing the survey, clinicians reported that there was no difficulty in recruiting people to deliver the ratings, as survey platforms (e.g., Surveymonkey; Qualtrics) and social media (e.g., Facebook) allow others (e.g., colleagues, friends) to be contacted to do so.
- Comparison experiments can become much more difficult to conduct under conditions of social isolation, where one might not see many people all day. Where such experiments (the impact of comparing your body to others' vs. not comparing your body to others'), then it can be valuable to present this as a naturalistic experiment (“how do you feel about your body now that you are not comparing it all day vs. when you used to do so?”). If the patient does a lot of body comparison on social media, then that can still be used as the basis of a controlled experiment, of course.
- Mirror exposure remains possible when working with a video link, though it requires careful positioning of the webcam without becoming a distraction. It is even more important to get the patient to do mirror exposure for homework between sessions, in order to maximize the dose.
  - An alternative approach that was suggested is for the patient to use their computer screen to show their image, while the therapist can also see it and can engage the patient in describing their body, detailing anxiety levels, etc. This is possible with some platforms (e.g., Google Meet) if the patient's image is “pinned” to the main screen. While this method can be harder to set up with a small screen, it is possible, and patients find it challenging in the short term (as with in‐person mirror exposure), but a good launch base for repeated exposure for homework.

*Working with core beliefs*. Negative core beliefs often underpin emotionally driven eating behaviors (e.g., binging to block emotions) and body image (e.g., where there is a trauma history). The clinicians note that:

- The majority of work with those core beliefs is cognitive, and the necessary exploration, formulation, historical review and attributional work can still be carried out remotely, as long as the patient is stable enough to tolerate the experience.
- Both imagery rescripting and chairwork/role play methods are still possible online.
- Encouraging counter‐schematic behaviors (e.g., mixing with other people) can be more challenging, though some of it can be achieved over the phone or online (e.g., addressing fears of abandonment by explaining true feelings to a friend).
- Environmental change might mean that there are fewer triggers to these emotional states (though loneliness and frustration might be more likely). This contextual difference gives clinicians the opportunity to stress that the core beliefs and emotions are situation‐specific, rather than being fundamental to the patient, thus helping with re‐attribution.

*Group work*. There were questions regarding whether group therapy could work online (e.g., would the group connect and feel safe). The experience of a large number of clinicians was that:

- Patients find that online groups are effective under these conditions.
- Clinicians who were previously running groups online were finding that such groups were no more or less effective than they had been before the pandemic conditions set in.
- There were positive comments about the experience of running online groups for binge‐eating disorders and for low‐weight adolescents.

*Post‐session contact*.

- Consider emailing a summary to the patient after the session, summarizing what has been covered, what the plan is, and the broad agenda for next time.

### 3.7 Attention to local regulatory frameworks

Of course, all of the above should be considered within the regulatory frameworks that apply to all telehealth and data sharing. These will be set by employers (e.g., what platforms can be used), professional bodies such as the American Psychological Association—see Appendix A, and governments (e.g., data protection). These frameworks are there to protect the therapist and patient alike, and all psychotherapists should be observing them to ensure safe and good practice.

## 4 DISCUSSION

This article is a summary of the ideas that emerged from clinicians who took part in an online approach to CBT‐ED in the context of the Coronavirus on patients, clinicians and services. It is provided so that we can respond helpfully to this pandemic. We note that some of the ideas are specific to the restrictions inherent to the pandemic, which has resulted in many therapists working from home, introducing unique technical, logistical, and psychological challenges. Under more normal circumstances, telehealth would normally be conducted from the therapist's workplace. It is in no way a scientifically robust paper, having been based on a limited sample and the ideas that were expressed in the first 96 hr of sharing ideas and experiences. We encourage clinicians to visit the relevant Google sheet to identify new ideas that have been added since then, and to contribute their own.

We hope that these suggestions support clinicians in their innovative use of CBT‐ED, but we found that many of the suggestions could be applied to all therapies. In future, it would be useful to undertake similar studies of the application of other therapies for eating disorders under such unusual circumstances, using more structured methods (e.g., Delphi approaches) than were possible in this short timeframe. Similarly, it will be valuable to compare these conclusions about CBT‐ED with recommendations that emerge for the treatment of other disorders, to establish common lessons across disorders as well as therapies. Of course, there are clinicians who are well‐versed in the delivery of telehealth, for whom these conclusions might be seen as relatively obvious, and we welcome their supportive contribution to the suggestions outlined here. However, while there is some recent preliminary evidence that telehealth can be effective in FBT for adolescents with anorexia nervosa (Anderson et al., 2017), many clinicians have found this transition to be a new experience, and that has led to the need to think about transitions and future practice.

Such work might be seen as running the risk of taking us away from evidence‐based protocols (e.g., Fairburn, 2008; Waller et al., 2019), but we would argue that this approach is simply using the flexibility to the individual patient's needs that ought to be seen as inherent in such protocols (Wilson, 1996). Existing telehealth methods have already developed some evidence for this approach, though not under these exceptional circumstances. The only way of knowing whether these clinical recommendations are useful is to try them out and to evaluate the outcome. We suggest that clinicians should use their existing data collection methods to compare their patients' outcomes across cohorts based on patients who were treated face‐to‐face before the current COVID‐19 pandemic, patients treated entirely by telehealth methods during this period, and (possibly most interesting) those whose treatment modality was forced to change during therapy as a result of the changes in healthcare provision. We also recommend online supervision to keep therapists on track with the delivery of protocols.

In the short term, we hope that therapists will learn enough from these clinical recommendations to be potentially more flexible in their delivery of evidence‐based therapies, particularly in the context of any future disruptions to normal service delivery. However, this exercise in sharing information and developing consensus in a relatively short time frame also has longer term benefits. At a later stage, it is also possible that what we learn from these responses to the current crisis might teach us to be more effective in delivering telehealth in routine practice, enhancing the accessibility of effective treatment for eating disorders when normal service is resumed.

## CONFLICT OF INTEREST

The authors declare no conflicts of interest.

## Data Availability

The data used are available on the original online form: https://docs.google.com/document/d/1n5X1zC_4lHMUH3V0JF8ZvEWhKFTZrjKzUYo6DwxPrco/edit.

## Appendix A Resources identified to assist clinicians and patients

*Google sheet detailing the topics raised and suggestions made*:

https://docs.google.com/document/d/1n5X1zC_4lHMUH3V0JF8ZvEWhKFTZrjKzUYo6DwxPrco/edit

*Existing guidelines about delivering psychotherapy via telehealth*:

https://www.apa.org/practice/guidelines/telepsychology

https://www.nationalregister.org/npc-telepsych-video/

https://www.crpo.ca/implementing-electronic-practice/

*Online measures, diaries, psychoeducation materials, etc*.:

Centre for Clinical Interventions—https://www.cci.health.wa.gov.au/Resources/Looking‐After‐Yourself/Disordered‐Eating (including fillable pdf forms that can easily be returned online).

CREDO site—https://www.credo-oxford.com/4.4.html

BEAT—https://www.beateatingdisorders.org.uk/coronavirus

NEDIC—https://nedic.ca/covid-19-ed-faqs

CBT‐T website—http://cbt-t.group.shef.ac.uk/

*Eating to support the immune system*:

https://www.eatright.org/health/wellness/preventing‐illness/how‐to‐keep‐your‐immune‐system‐healthy

*COVID‐19 specific dietary advice*:

https://www.bda.uk.com/resource/covid‐19‐corona‐virus‐advice‐for‐the‐general‐public.html

*Example of quarantine food plans*:

e.g., https://www.recipetineats.com/coronavirus-menu-plan-1

*Professional bodies' toolkits and courses for newly remote workers and the delivery of telehealth*:

https://www.apa.org/news/apa/2020/03/newly‐remote‐workers

https://www.apa.org/education/ce/telehealth‐001 (currently a free course)

https://www.psychiatry.org/psychiatrists/practice/telepsychiatry/toolkit

https://education.psychiatry.org/

https://education.smiadviser.org/Users/ProductDetails.aspx?ActivityID=7257

## Appendix B Information sheet for patients undertaking online CBT‐ED

### Online therapy for eating disorders

Coronavirus has led to changes in the way that mental health services deliver talking therapies. In order to limit face‐to‐face contact, many services now provide therapy online (sometimes exclusively). This information sheet explains how internet‐based (cognitive behavioral therapy for eating disorders [iCBT‐ED]) (brief cognitive behavioral therapy for eating disorders [iCBT‐T]) is delivered and how you can prepare for the start of your treatment.

We are aware that online therapy may not be your preferred method of treatment or the treatment that you originally agreed to. However, it is important that face‐to‐face contact is limited in order to protect your health and the health of others. Given that the process and content of [iCBT‐ED/iCBT‐T] is no different to face‐to‐face therapy, there is no reason that it should be less effective than therapy delivered in person.

### Structure of iCBT‐ED/CBT‐T

iCBT‐ED/T for [DIAGNOSIS] is provided over [NUMBER] sessions. Your treatment will be reviewed [REGULARLY/AT SESSION x]. Sessions are provided on a weekly basis and will last approximately 50 min. Attending sessions consistently are vital to your treatment being effective.

Your therapist will contact you at the specified time using [PLATFORM]. It is important that you are ready to meet at the agreed time.

Please be aware that your therapist cannot be contacted using [PLATFORM] outside of your appointments.

### Preparing for your sessions

*What you will need*:

You will need a pen and paper for each your sessions. Monitoring your weight plays an important role in CBT‐ED/T. For this reason, you also need access to weighing scales during your appointments. Your therapist will discuss this with you in more detail during your first meeting.

*Software*:

You will be using [PLATFORM] for your therapy sessions. It is a good idea to practice using [PLATFORM] before your first session so that you are familiar with how it works. This also ensures that your software is up‐to‐date.

*Hardware*:

It can be difficult to focus on therapy sessions if you are using a small mobile phone screen which is easily moved. We recommend using either a laptop or desktop computer. If you only have a phone or tablet, please make sure it is on a stable stand.

*Connectivity*:

Poor internet connection can disrupt online therapy sessions. Prior to your session, make sure that you are somewhere that your access to the internet is strong and reliable. This may mean finding a place that is close to your internet router.

*Location*:

It is important that you find a space that feels safe, comfortable, and will not be disturbed during your therapy sessions. If possible, find a place that you can use throughout the course of your treatment. Public places and talking while driving are not recommended.

*Privacy*:

It is important that your sessions are private and confidential. Find a location where you will not be interrupted, and you are able to speak freely. If needed, let the individuals around you know that they should not disturb you for the duration of your meeting. You may find it helpful to use headphones or a headset during your sessions if others are nearby.

*Distractions*:

Try to limit things that might distract you during your sessions. These might include the TV or radio, nearby conversations, phone calls, noisy animals, drinking, or smoking. Remember that your therapist will need to do the same if working from home.

### Contact between appointments

Contact between your therapy sessions is limited. Your therapist may email you between appointments for the following reasons:

- To summarize your session.
- To send you resources discussed during the appointment.
- To send appointment confirmations or notify you of appointment changes.

Your contact with your therapist should focus on key tasks of therapy, such as sending them copies of your homework prior to your next session. Please remember that therapy for your eating disorder should be going on all week, so the work you do between sessions is really vital, and you should discuss problems and how you solved them during your therapy sessions.

*Matthew Pugh*

*With thanks and credit to Conor O'Brien, Lauren Antinoro, and Xi Liu*
